# Evaluating Nitrogen Management Practices for Greenhouse Gas Emission Reduction in a Maize Farmland in the North China Plain: Adapting to Climate Change

**DOI:** 10.3390/plants12213749

**Published:** 2023-11-02

**Authors:** Huayun He, Qi Hu, Feifei Pan, Xuebiao Pan

**Affiliations:** 1 College of Resources and Environmental Sciences, China Agricultural University, Beijing 100193, China; 13716980016@163.com (H.H.); panxb@cau.edu.cn (X.P.); 2 China Meteorological Administration-China Agricultural University (CMA-CAU) Jointly Laboratory of Agriculture Addressing Climate Change, Beijing 100193, China; 3 Department of Geography and the Environment, University of North Texas, Denton, TX 76203, USA

**Keywords:** maize yield, nitrogen management, life cycle assessment, greenhouse gas, agricultural net profit

## Abstract

Quantification of the trade-offs among greenhouse gas (GHG) emissions, yield, and farmers’ incomes is essential for proposing economic and environmental nitrogen (N) management strategies for optimizing agricultural production. A four-year (2017–2020) field experiment (including four treatments: basic N fertilizer treatment (BF), suitable utilization of fertilization (SU), emission reduction treatment (ER), and high fertilization (HF)) was conducted on maize (*Zea mays* L.) in the North China Plain. The Life Cycle Assessment (LCA) method was used in this study to quantify the GHG emissions and farmers’ incomes during the whole maize production process. The total GHG emissions of BF, SU, ER, and HF treatments in the process of maize production are 10,755.2, 12,908.7, 11,950.1, and 14,274.5 kg CO_2_-eq ha^−1^, respectively, of which the direct emissions account for 84.8%, 76.8%, 74.9%, and 71.0%, respectively. Adding inhibitors significantly reduced direct GHG emissions, and the N_2_O and CO_2_ emissions from the maize fields in the ER treatment decreased by 30.0% and 7.9% compared to those in the SU treatment. Insignificant differences in yield were found between the SU and ER treatments, indicating that adding fertilizer inhibitors did not affect farmers’ incomes while reducing GHG emissions. The yield for SU, ER, and HF treatments all significantly increased by 12.9–24.0%, 10.0–20.7%, and 2.1–17.4% compared to BF, respectively. In comparison with BF, both SU and ER significantly promoted agricultural net profit (ANP) by 16.6% and 12.2%, with mean ANP values of 3101.0 USD ha^−1^ and 2980.0 USD ha^−1^, respectively. Due to the high agricultural inputs, the ANP values in the HF treatment were 11.2%, 16.6%, and 12.4% lower than those in the SU treatment in 2018–2020. In conclusion, the combination of N fertilizer and inhibitors proved to be an environmentally friendly, high-profit, and low-emissions production technology while sustaining or even increasing maize yields in the North China Plain, which was conducive to achieving agricultural sustainability.

## 1. Introduction

Modern agricultural production is an energy- and carbon-intensive process [1] releasing tremendous greenhouse gases (GHGs) into the atmosphere [2]. Agriculture is one major contributor to anthropogenic carbon dioxide emissions and caused a 35% increase in global GHG emissions during 1970–2010 [3,4]. China’s agricultural development is crucial to national food security and social stability; however, it also impacts global carbon emissions that cannot be ignored. In China, GHG emissions caused by agricultural activities contributed approximately 17–32% of national GHG emissions [5,6]. The farming-associated GHGs released into the atmosphere are derived from direct and indirect emissions. In the process of crop production, N_2_O is directly emitted by the application of nitrogen fertilizer in the field. In addition, the energy consumption in the production and transportation of the inputs (seeds, fertilizers, and herbicides) for farming leads to GHG emissions, as well as the energy consumption in the field management (tillage, irrigation, sowing, and harvesting) [7,8]. Therefore, how to balance agricultural carbon emissions and crop yields has become a critical issue for reducing energy consumption and improving agricultural sustainability.

The North China Plain is a crucial maize-producing region in China and holds 22.89% of the total national cultivated land [9]. To obtain high crop yields, nitrogen fertilizer has long been used, far exceeding the demand for crops in the North China Plain. For example, more than 600 kg N ha^−1^ yr^−1^ was used for the annual wheat-maize system in some areas [10]. However, such practices did not significantly improve crop yield but doubled the amount of N released into the environment [11]. Excessive fertilization reduces the nitrogen use efficiency (NUE) of crops and causes higher CO_2_ and N_2_O emissions from soils [12,13,14]. Promoting cleaner agricultural practices with less fertilizer and less environmental pollution is imperative to achieving sustainable agricultural development in China. Site-specific management of nitrogen fertilizer has the potential to reduce nitrate pollution while reducing economic variability and maintaining profitability [15]. It has become increasingly evident that optimizing the fertilizer type has positive effects on the improvement of environmental quality and the ideal balance for economic profitability. Using controlled-release nitrogen fertilizer, biochar, organic fertilizer, and microbial inoculum could effectively enhance crop yields and improve N use efficiency and the effectiveness of fertilizer in agroecosystems [16,17,18,19]. Chemical nitrification inhibitors (NI) and urease inhibitors (UI) have been proven effective in reducing the loss of N from soils. Wheat straw mulching with NI application achieved better balance among agronomic, economic, and environmental benefits for dryland maize in northwest China [20].

Although the application of inhibitors to reduce N_2_O emissions has been well reported in many areas [21,22], inconsistent results have been obtained [23,24,25,26], especially the comprehensive impact of the combined use of NI and UI on the agronomic, economic, and environmental benefits of summer maize farmland in the North China Plain, which has not been fully investigated. To fill this knowledge gap, based on the Life Cycle Assessment (LCA) [27], a 4-year field experiment with different nitrogen management practices was conducted in the North China Plain to test the following hypotheses: (i) suitable fertilizer rates with NI and UI can reduce GHG emissions in the maize growth process; (ii) suitable fertilizer rates with NI and UI represent a feasible pathway for maintaining yield while minimizing GHG emissions and maximizing ANP.

## 2. Results

### 2.1. GHG Emissions Analysis

#### 2.1.1. Direct Emissions

**Figure 1:**
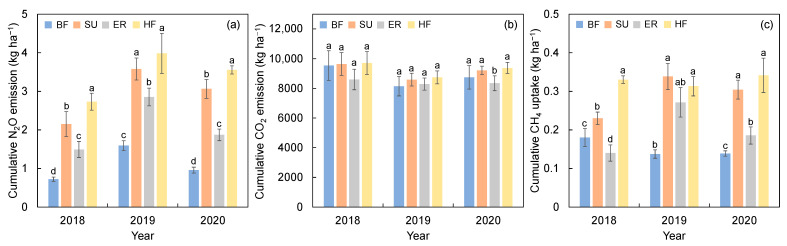
Cumulative emission/uptake quantities of N_2_O (**a**), CO_2_ (**b**), and CH_4_ (**c**) under different fertilizer treatments during the maize growing seasons in 2018, 2019, and 2020. BF: basic N fertilizer treatment; SU: suitable utilization of fertilization; ER: emission reduction treatment, same fertilization as SU with a nitrification inhibitor and urease inhibitor; HF: higher fertilization than SU. The vertical bars represent the LSD value (*p* = 0.05). Different letters in the inset denote significant differences (*p* < 0.05) in cumulative N_2_O, CO_2_, or CH_4_ fluxes among treatments.

In comparison with the N_2_O emissions under BF, the total N_2_O emissions of the SU, ER, and HF treatments increased by 107.8%, 62.1%, and 143.1%, respectively (Figure 1a). The HF treatment produced the highest N_2_O emission, reaching 2.73 kg ha^−1^, 3.98 kg ha^−1^, and 3.56 kg ha^−1^ in 2018, 2019, and 2020, respectively (Figure 1). Nitrification inhibitors and urease inhibitors significantly decreased the N_2_O emission, with the annual N_2_O emission of ER treatment being 30.0% less than that of SU treatment.

Unlike N_2_O, there is no significant difference in the cumulative CO_2_ emissions among the different treatments. From 2018 to 2020, the range of the cumulative CO_2_ emissions in each year was 8594.9–9700.2, 8138.9–8742.5, and 8341.5–9371.3 kg ha^−1^, respectively. Annual CO_2_ emissions under the ER treatment were 7.9% less than those under the SU treatment, and such an insignificant difference indicates that the addition of inhibitors had no significant effect on CO_2_ emissions. Under the HF treatment, CO_2_ emissions were highest in 2018, 2019, and 2020, at 9700.2 kg ha^−1^, 8742.5 kg ha^−1^, and 9371.3 kg ha^−1^, respectively, 5.3% higher on average than the BF treatment (Figure 1b).

Within 3 years, the absorption of CH_4_ by the HF treatment was 0.33 kg ha^−1^, 0.31 kg ha^−1^, and 0.34 kg ha^−1^, respectively, which were 1.8, 2.3, and 2.5 times that of the BF treatment. Average CH_4_ uptake under the ER treatment was 32.6% lower than the SU treatment in maize growing seasons.

#### 2.1.2. Total GHG Emissions

**Figure 2:**
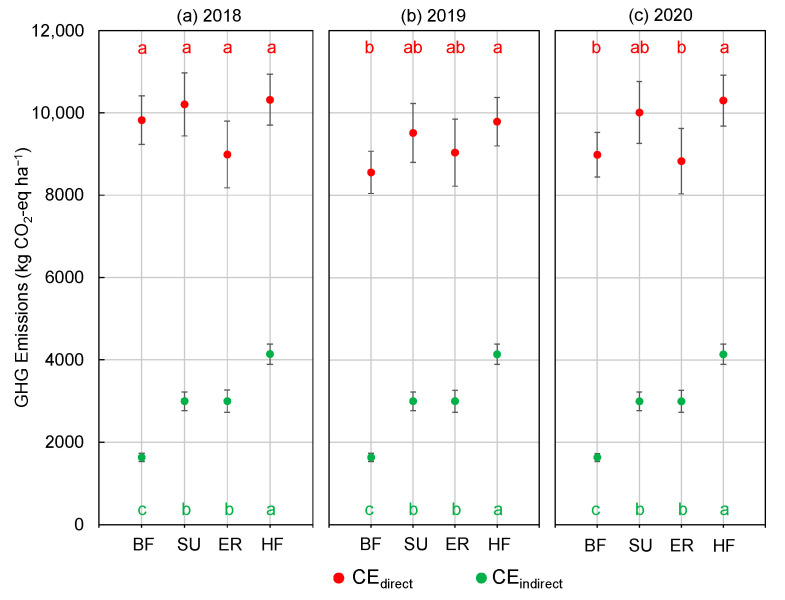
The direct and indirect GHG emissions of maize production in 2018 (**a**), 2019 (**b**), and 2020 (**c**). BF: basic N fertilizer treatment; SU: suitable utilization of fertilization; ER: emission reduction treatment, same fertilization as SU with a nitrification inhibitor and urease inhibitor; HF: higher fertilization than SU. The vertical bars represent the LSD value (*p* = 0.05). Different letters in the inset denote significant differences (*p* < 0.05) in direct and indirect GHG emissions among treatments.

According to the different GHG emission sources, the average direct and indirect emissions for maize production were computed, and it was found that the direct GHG emissions were the main contributors (Figure 2). The direct GHG emissions of crop growth accounted for 84.8%, 76.8%, 74.9%, and 71.0% of total GHG emissions for BF, SU, ER, and HF, respectively, with mean values of 9121.5 kg CO_2_-eq ha^−1^, 9910.2 kg CO_2_-eq ha^−1^, 8951.5 kg CO_2_-eq ha^−1^, and 10,135.7 kg CO_2_-eq ha^−1^ (Figure 2). Among the direct GHG emissions, the total CO_2_ emissions were the highest, and CH_4_ served as a carbon sink in the agricultural ecosystem. The farm management practices and production of various agricultural materials produced smaller GHG emissions, ranging from 1663.7 kg CO_2_-eq ha^−1^ (BF) to 4138.8 kg CO_2_-eq ha^−1^ (HF) in 2018–2020.

The HF treatment produced significantly higher emissions than BF in 2018–2020, with the direct and indirect GHG emissions increasing by 11.1% and 153.3%, respectively (Figure 2). Adding inhibitors significantly reduced direct emissions, i.e., in comparison to SU, the total emission for ER treatment decreased by 3.8–9.2% in 2018–2020, mainly resulting from the decreases in N_2_O.

### 2.2. Yield and Its Components

Table 1 shows the yield and yield components of summer maize under different treatments from 2017 to 2020. During the 4-year trial, the maize yield in this study area ranged from 9212 kg ha^−1^ kg ha^−1^ to 13,005.6 kg ha^−1^; SU, ER, and HF treatments all significantly increased the minimum and maximum maize yields by 12.9–24.0%, 10.0–20.7%, and 2.1–17.4% compared to BF, respectively (Table 1). BF exhibited the lowest number of kernels per ear, while insignificant differences for the number of ears and 1000-kernal weight were found among the four treatments. The yield for SU, ER, and HF treatments had no significant differences in all four years, as well as the ear number, number of kernels per ear, and 1000-kernal weight (Table 1). Compared to SU, the maize yield in the ER treatment was slightly reduced in most years, but those differences were not significant, indicating that adding nitrification inhibitors and urease inhibitors had no effect on the maize yield under the same nitrogen application level.

### 2.3. Economic Profits Analysis Based on GHG Emission and Yield

**Figure 3:**
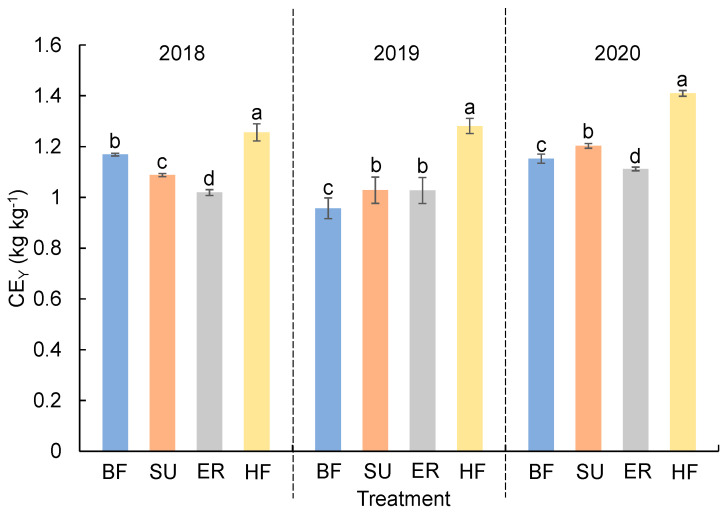
The CE_Y_ under different fertilizer treatments of maize production in 2018–2020. BF: basic N fertilizer treatment; SU: suitable utilization of fertilization; ER: emission reduction treatment, same fertilization as SU with a nitrification inhibitor and urease inhibitor; HF: higher fertilization than SU. The vertical bars represent the LSD value (*p* = 0.05). Different letters in the inset denote significant differences (*p* < 0.05) in CE_Y_ among treatments.

The CE_Y_ ranges of different treatments were 1.02 kg kg^−1^–1.26 kg kg^−1^, 0.95 kg kg^−1^–1.28 kg kg^−1^, and 1.11–1.41 kg kg^−1^ in 2018, 2019, and 2020, respectively (Figure 3). The ER treatment could decrease CE_Y_ by 4.7% compared to SU during 2018–2020, which indicated that the ER planting system emitted fewer GHGs per unit maize production. Results also showed that there were no significant CE_Y_ differences between ER and BF, and the CE_Y_ of HF treatment was significantly improved by 21.2% compared to BF.

**Figure 4:**
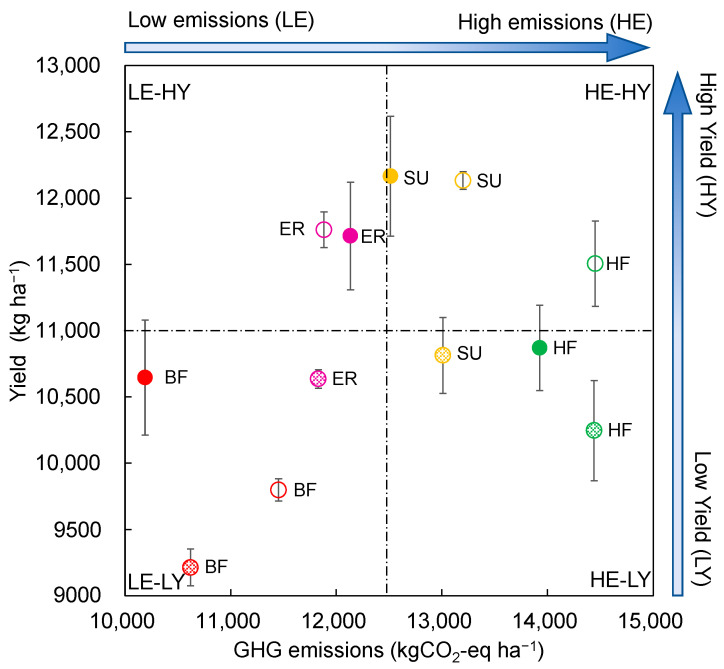
Classification of different nitrogen management methods based on GHG emissions-maize yield. Low emissions (LE): 10,000 < GHG emissions < 12,500; high emissions (HE): 12,500 < GHG emissions < 15,000; low yield (LY): 9000 < yield < 11,000; high yield (HY): 11,000 < yield < 13,000. The hollow circle, solid circle, and slant-filled circle represent the GHG emissions and maize for 2018, 2019, and 2020, respectively. The vertical bars represent the LSD value (*p* = 0.05).

The differences in the ANP and GHG emissions for all treatments were further explored, and four emission–profit modes were defined (Figure 4), i.e., high emission–high yield (HE–HY), low emission–high yield (LE–HY), high emission–low yield (HE–LY), and low emission–low yield (LE–LY). Compared to BF (LE–LY), the HF treatments showed HE–LY mode, i.e., higher GHG emissions and lower maize yield, indicating that the high nitrogen application rate in HF should be avoided in the maize production in this study area. Although the SU treatment could lead to a high yield, it produced higher GHG emissions compared with BF, and thus this treatment is not a proper measure for green and low-carbon development. As exhibiting a LE–HY mode, the ER treatment’s fertilization management can be used for demonstration and promotion of efficiency and emission reduction technology for summer maize planting and production in the North China Plain.

**Figure 5:**
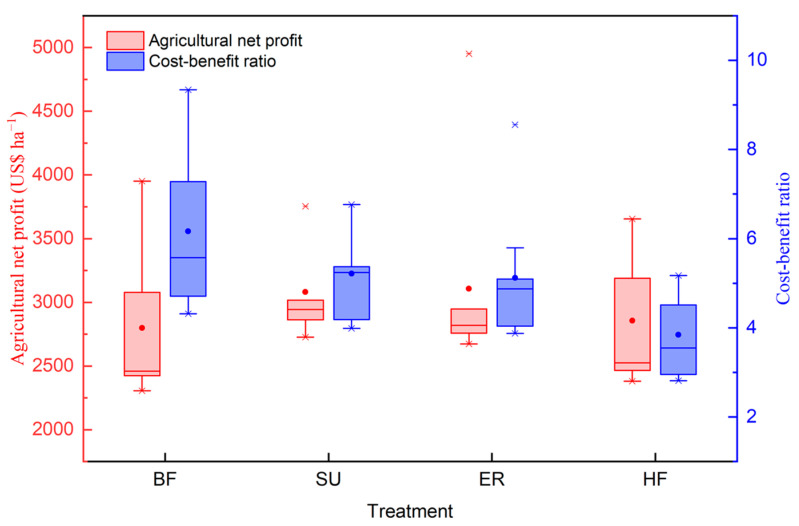
Maize agricultural net profit and cost-benefit ratio under different treatments in the North China Plain. BF: basic N fertilizer treatment; SU: suitable utilization of fertilization; ER: emission reduction treatment; same fertilization as SU with a nitrification inhibitor and urease inhibitor; HF: higher fertilization than SU. The solid line in the box represents the median value, the box boundary indicates the 25th and 75th percentiles, and dots indicate the average value.

The agricultural outputs in all treatments in 2018–2020 were higher than the costs of agricultural inputs, with the ANP ranging from 2415.0 US$ to 3542.7 US$ (Figure 5). In comparison to BF, both SU and ER significantly promoted ANP by 16.6% and 12.2%, respectively, and the mean benefits were 3101.0 US$ and 2980.0 US$, respectively. In contrast, due to the high agricultural inputs, the ANP values in the HF treatment were significantly lower than those in SU: 11.2% lower in 2018, 16.6% lower in 2019, and 12.4% lower in 2020.

The average value of the cost-benefit ratio ranged from 4.6 to 6.9 for the four treatments in this study area (Figure 5). The cost-benefit ratios of SU and ER were lower than those achieved under the BF, and the decreases compared to the BF were 8.4% and 13.3% in 3 years, respectively. Compared to BF, the cost-benefit ratio in the HF treatment was reduced by 28.2% in 2018, 38.9% in 2019, and 29.8% in 2020. Results also showed that there were no significant differences in ANP and cost-benefit ratio between the SU and ER treatments, indicating that adding fertilizer inhibitors did not affect farmers’ income while reducing emissions.

## 3. Discussion

### 3.1. Soil Gases Emissions

#### 3.1.1. N_2_O Emission

In order to achieve high grain yields in the North China Plain, excessive nitrogen fertilizer had been applied in the fields and resulted in the loss of a large number of N elements through N_2_O emissions, nitrogen leaching, and ammonia volatilization, which increased GHG emissions. The results showed that the application of nitrogen fertilizer was the main factor affecting N_2_O emissions from farmland soils [28]. Soil moisture is the main driving factor for N_2_O emissions, as it regulates the oxygen availability of soil microbes. Fertilization provides a nitrogen source for the release of N_2_O. After chemical nitrogen fertilizer is applied to the soil, nitrite nitrogen is transiently accumulated due to nitrification, thus providing a substrate for the denitrification process of nitrifying bacteria. Rainfall increases soil moisture, promotes soil organic matter mineralization, and provides the reaction substrate NH_4_^+^ for nitrification. At the same time, the nitrification product NO_3_^−^ is also the reaction substrate of denitrification, and the synergistic occurrence of the two processes promotes the formation of N_2_O. The increase in soil moisture also enhanced the activity of N_2_O-producing microorganisms, which was conducive to N_2_O production.

Adding urease and nitrification inhibitors to nitrogen fertilizer is an important measure to reduce N_2_O emissions and increase crop yield [29,30]. Urease inhibitors can block the reaction process of urea hydrolysis to ammonium carbonate and NH_3_ formation by urease and prolong the time of urea entering the soil. Nitrification inhibitors mainly delay the conversion of ammonium ions (NH_4_^+^) into NO_3_^−^, decrease the nitrification rate, and reduce nitrogen volatilization (such as nitrogen oxides) or nitrogen leaching (such as NO_3_^−^ or NO_2_^−^), thus limiting N_2_O emissions from soil denitrification.

Previous studies have found that environmental conditions (precipitation and temperature) could significantly affect the GHG emissions from the farmland [31,32,33]. We further compared the N_2_O emissions in 2018 (the dry year with 300.89 mm in the maize growing season) and 2019 (the wet year with 555.79 mm in the maize growing season) and found that the total N_2_O emissions were 69.3% higher in the wet year than the dry year, which was consistent with the results in Ju et al. [34].

#### 3.1.2. CO_2_ and CH_4_ Emissions

In this study, the measured CO_2_ fluxes were expressed as soil respiration. Soil respiration (R_s_, CO_2_ efflux) is a major flux of CO_2_ into the atmosphere. R_s_ originates from rhizospheric respiration (R_a_) arising from root and rhizomicrobial respiration and rhizosphere priming effects, and heterotrophic respiration (R_h_) by microbes decomposing plant and root detritus and soil organic matter (SOM). At present, the effect of N addition on soil respiration is inconsistent in the literature. The application of nitrogen fertilizer has been proven to reduce root carbon allocation and rhizosphere microbial respiration, inhibit fungal activity, and lead to reduced decomposition, synergistically reducing carbon dioxide flux [35,36]. In contrast, other studies have reported that N additions stimulate soil respiration by accelerating soil organic matter decomposition. The experimental results of Jassal et al. [37] showed that N-fertilization initially caused a significant increase in R_s_ and R_h_. Cleveland and Townsend [38] found that N addition resulted in increased Rs in the field, attributed to the promotion of fine root growth by N fertilization application. In this study, our results indicated that the application of nitrogen fertilizer generally had no significant effects on CO_2_ emissions because there was no significant difference in CO_2_ cumulative emissions among the different treatments. The higher CO_2_ emissions of the HF treatment in this study may be due to the increase in the amount of chemical fertilizer, which directly provides the nitrogen required for the growth of plants and microorganisms, resulting in more developed crop roots left in the field in the treatment with high fertilizer application. On the other hand, N affects microbial activity by directly affecting soil pH levels. Seasonal dynamics of soil CO_2_ flux followed air and soil temperature variations, crop growth speed, and respiration rate in three years [39].

Methane-oxidizing bacteria in dryland soils could consume and absorb CH_4_ from the air near the ground and use CH_4_ as the sole carbon source [40]. Nitrogen application inhibited CH_4_ uptake, but the addition of inhibitors increased nitrogen content in the soil, which was not conducive to the survival of methane-oxidizing bacteria and significantly reduced CH_4_ absorption. Compared with the SU treatment, the uptake of CH_4_ in the ER treatment decreased by 32.6%. During the summer maize growing season, there was no clear mechanism of CH_4_ uptake by the soil. The effect mechanism of fertilization on CH_4_ emission flux is complex. Increasing nitrogen fertilizer promoted the proliferation of methanogens but inhibited the growth of methane-oxidizing bacteria. Therefore, except for ER, there was no significant difference in the CH_4_ emission flux of the other treatments.

### 3.2. Direct Carbon Emissions

The promotion effect of the recommended field management practices on balancing high grain yields and environmental benefits has been clearly revealed. The use of nitrogen fertilizer has caused a great waste of resources for a long time. There are several ways that a maize production system could reduce direct GHG emissions. Solid granular urea has been the most heavily applied N fertilizer in farmland in the maize production region of the North China Plain. Controlled-release fertilizer can provide progressive nutrient supply to crops, and if it is mixed with urea, it can significantly reduce the warming potential (21.1%) and carbon footprint (21.7%) and improve the economic efficiency of maize (4.9–12.1%) [41]. Our study shows that N_2_O emissions from the ER treatment are reduced by an average of 30% compared to the SU treatment, and total emissions are reduced by 3.8–9.2% in 2018–2020. The reason that the reduction effect of adding inhibitors is slightly lower than that of combining controlled-release fertilizer with urea is that we included CO_2_ emissions in the calculation of total emissions, which reduces the reduction effect. However, the effectiveness of controlled-release fertilizer is subject to certain limitations, such as local climate, soil type, soil moisture conditions, and high prices. Straw returning to the field can improve soil fertility, but its relationship with greenhouse gas emissions is not clear. Some studies have shown that returning straw to the field provides available C and N to the soil, stimulates the activity of soil microorganisms, and thus promotes soil greenhouse gas emissions [42,43]. On the contrary, some studies have shown that greenhouse gas emissions will be inhibited if a reasonable straw-returning model is adopted [31,41,44]. There are very few studies on the influence of climate change and soil types on straw returns in the North China Plain. Compared with straw returning, adding inhibitors is a more direct and effective way to reduce greenhouse gas emissions. In addition, Sosulski et al. [45] evaluated the effects of fertilization methods on soil N_2_O and CO_2_ emissions and found that the effect of deep application of nitrogen fertilizer on reducing soil N_2_O emissions was completely offset by the increase in soil CO_2_ emissions, but their study area was sandy soil without crop cover. The soil type of the North China Plain is tidal soil, and the effect of deep application of nitrogen fertilizer on reducing greenhouse gas emissions may be considerable. In conclusion, the effect of nitrogen fertilizer on soil greenhouse gases is a complex problem, and it is necessary to continue to explore the cleaner production methods that are more suitable for corn production in the North China Plain in the future.

### 3.3. Indirect Carbon Emissions

In recent years, there have been controversies about the types of GHGs and the system boundaries of the carbon budget [46,47]. Therefore, in many studies of the estimation of carbon emissions from food crop productions, the settled results of maize are quite different, with great uncertainty. The contribution of material and energy inputs to GHGs cannot be ignored. It is an incomplete evaluation of the carbon budget in the agricultural system without considering the emission flux of CO_2_ to the atmosphere caused by energy consumption. In particular, the impact of nitrogen fertilizer as the largest energy consumption input in agricultural production on CO_2_ emissions should not be ignored [48]. Chemical fertilizer and irrigation were the largest contributors to GHG emissions in agricultural fields [6]. Compared with the energy production of some other developed countries, coal combustion is the major energy source for agriculture in China. However, the energy conversion efficiency of coal is usually low, which indicates that CO_2_ emissions from agricultural production in China would be higher than those in other developed countries. Chen et al. [49] calculated the GHG emission coefficients (from raw materials to factory gates) in the manufacturing process of various nitrogen fertilizers, phosphate fertilizers, and potash fertilizers in line with China’s current situation by collecting and integrating relevant domestic data. The GHG emissions coefficient of nitrogen, phosphorus, and potassium fertilizers is generally about twice that of the European and American averages. Therefore, the use of foreign coefficients to estimate China’s agricultural GHG emissions will seriously underestimate the impact of chemical fertilizer application. In this study, the carbon emissions from chemical fertilizer accounted for more than 70% of the carbon emissions from agricultural production. Using the life cycle assessment method, Zhang et al. [50] estimated that the GHG emissions of nitrogen fertilizer production, processing, and transportation in China are as high as 8.3 kg CO_2_-eq, which is 60% higher than the greenhouse effect caused by GHG emissions caused by field application. Some studies suggested that CH_4_ accounted for a small proportion of GHG emissions in dryland, so it should not be included in the calculation. Soil N_2_O emissions have not been calculated either. In this study, CO_2_, NO_2_, and CH_4_ released indirectly by agricultural input were included in the analysis, and the carbon balance of different fertilization treatments was comprehensively and systematically analyzed.

### 3.4. Effects of N Management on Yield and Economic Profits

To pursue high grain yields, the farmers in the North China Plain prefer excessive application of nitrogen fertilizer. However, this study found that no higher yield was obtained with the high fertilizer rate in the HF treatment, and ironically, the farmers’ income decreased due to the higher input. As Nitrogen fertilizer rate was the main factor affecting N_2_O emissions from farmland soils [28], significant increases (143.1% higher than BF) of the GHG emissions in the HF treatment were found in this study. Tan et al. [51] also concluded through field experiments that a 30% reduction in nitrogen input in a winter wheat–summer maize rotation system can significantly reduce total GHG emissions while maintaining grain yield. Wu et al. [52] found that if maize production areas in China take optimized nitrogen fertilizer measures, 1.4 million tons of nitrogen fertilizer and 18.6 million tons of GHG emissions can be reduced each year. Adding nitrification inhibitors and urease inhibitors was reported as an effective method to reduce GHG emissions [29,30], and this study exhibited that the N_2_O and CO_2_ emissions in the ER treatment were both decreased by 30.0% and 7.9% under the same nitrogen application rate. Meanwhile, insignificant yield differences were found between the ER and SU treatments, which was consistent with previous research results [53,54]. ER has two characteristics, i.e., low GHG emissions and high ANP. While reducing GHG emissions, ER significantly improved the net income of maize, which is in line with the connotation core of low-carbon modern agriculture. Therefore, controlling the application rate of N fertilizer is necessary to reduce both direct and indirect GHG emissions.

In this study, since only one nitrification inhibitor and one urease inhibitor were used, it is necessary to further explore the effects of the mixed application of nitrogen fertilizer, NIs, and UIs, on reducing emissions and increasing economic profits. In summary, as for the maize production in the North China Plain, using fertilizer inhibitors was a green agriculture method because of low GHG emissions and high crop income, while applying high fertilization was the opposite measure, leading to high GHG emissions and low crop income. To achieve carbon neutrality and effectively adapt to climate change, we highly recommend that agricultural practices with low GHG emissions and high crop income be used and promoted in China.

## 4. Materials and Methods

### 4.1. Experimental Sites

**Figure 6:**
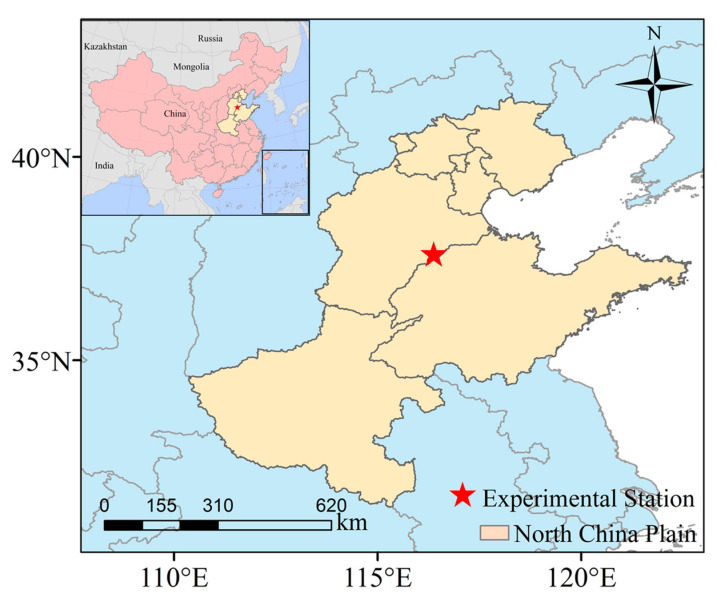
Map showing the location of the experimental station and study area.

**Figure 7:**
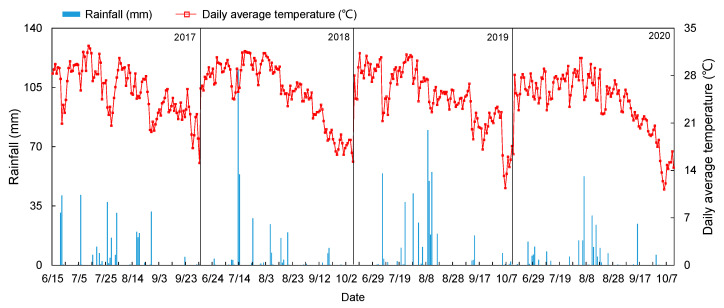
Precipitation and average temperature during the maize growth period of 2017–2020 at the Wu Qiao Experimental Station.

The North China Plain is the largest alluvial plain in China, including two province-level municipalities (i.e., Beijing and Tianjin) and three provinces (i.e., Hebei, Shandong, and Henan), with a total area of 536,628 km^2^ [55]. A four-year (from 2017 to 2020) field experiment was conducted at the Wuqiao Experimental Station (37.5° N, 116.4° E) in the North China Plain (Figure 6). Wuqiao County has a typical temperate monsoon climate, i.e., hot and rainy summers with an average temperature over 20 °C and cold and dry winters. The average annual total precipitation is about 500 mm, and 70% of rainfall occurs between June and September. Daily mean temperature and precipitation during the maize growing season in 2017–2020 are plotted in Figure 7. An automated weather station was installed in the experimental field to collect daily meteorological data. The soil type in the experimental area is fluvo aquic soil (Cambisols, FAO, Rome, Italy), with a pH value of 8.25. The content of soil organic matter, total nitrogen, ammonium nitrogen, alkali-hydrolyzable nitrogen, available phosphate, and available potassium in the 0–20 cm soil layer was 1.61%, 1.21 g kg^−1^, 7.80 mg kg^−1^, 80.18 mg kg^−1^, 29.57 mg kg^−1^, and 212.10 mg kg^−1^, respectively.

### 4.2. Experimental Design and Field Management

**Figure 8:**
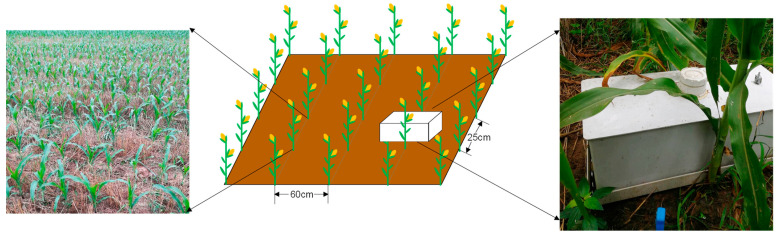
A schematic diagram for the layout of maize and static chamber locations. The white box represents the static chamber (0.6 m in length, 0.25 m in width, and 0.23 m in height), which was permanently installed between plants perpendicular to the planting row in the middle of the plot during the whole maize season. Each chamber could be matched to a frame base, which was inserted 10 cm into the soil at the beginning of the experiment, where it remained. The chamber was fitted into the frame base with a groove (5 cm width) during sampling, and the groove was sealed by infusing water between the chamber and the frame base.

The field experiment was conducted from 2017 to 2020 in maize (*Zea mays* L. Cv. Zhengdan 958) fields. The experiment had four treatments in a randomized block design with three replicates; each plot was 9.0 m long and 5.4 m wide. There were four treatments: (1) BF, basic N fertilizer treatment; (2) SU, suitable utilization of fertilization; (3) ER, emission reduction treatment with the same fertilization as SU but with a nitrification inhibitor and urease inhibitor; and (4) HF, higher fertilization than SU. The N type was urea (U, 46% N) for all treatments. The detailed NPK fertilizer usage for these treatments is shown in Table 2. Dicyandiamide (DCD, a nitrification inhibitor, content ≥ 98%) was manufactured by Wuxi Yatai United Chemical Co., Ltd., Wuxi, China, and hydroquinone (HQ, a urease inhibitor, content ≥ 98.5%) was manufactured by Shandong Baiqian Chemical Co., Ltd., Jinan, China. The fertilizer was applied just once on June 20 (ditch application between two rows of maize), and no extra fertilizer was applied during the maize growth period. After fertilization, irrigation was applied once (75 mm), and there was no other artificial replenishment during the experiment period. Manual weeding was performed throughout the experiment. All treatments had the same plant spacing (0.25 m) and row spacing (0.6 m), producing a planting density of 67,500 plants ha^−1^, which was consistent with the common practice of local farmers (Figure 8). Seeds were sown on 15 June of all years and harvested in early October in 2017–2020, and the average growth period of maize was 114 days.

### 4.3. Measurements and Calculation

#### 4.3.1. Crop Yield

At harvest, a 20 m^2^ area in the middle of each plot was manually harvested to determine the grain yield and yield components. The yield was expressed at 14% moisture content, according to the standard moisture for maize grain. The number of grains per spike and the 1000-grain weight were recorded.

#### 4.3.2. GHG Sampling and Measurements

The static chamber/gas chromatography method was used to measure soil CO_2_, CH_4_, and N_2_O emissions from 2018 to 2020. A PVC static chamber held by a frame was permanently installed between plants perpendicular to the planting row in the middle of each plot during the whole maize growing season (Figure 8). No plants or weeds existed on the soil surface covered by the collar to ensure that all the GHG collected was produced by the soil. The top edge of the collar had a groove, and the static chamber (0.6 m in length, 0.25 m in width, and 0.23 m in height) was placed with the rim of the chamber fitted into the groove while gas was sampled. The groove was filled with water to seal the rim of the chamber.

Four GHG samples were collected for each treatment between 09:00 and 11:00 a.m. since the soil temperature during the sampling period was close to the daily average soil temperature [56]. The first sample was taken immediately after the static chamber was placed, and the other samples were collected after 10, 20, and 30 min. Each GHG sample (80–120 mL) was taken from the static chamber using plastic syringes. The growth status of maize, soil moisture content, and temperature in the box were recorded simultaneously. Daily GHG samples were measured for seven consecutive days after fertilization, and then the samples were collected every ten days. An additional measurement was taken after each precipitation event.

The emission flux of N_2_O, CO_2_, and CH_4_ was calculated as follows [57]:

(1) $$F=\rho \times H\times \frac{\Delta C}{\Delta t}\times \frac{273}{273+T}\times k$$

where F is the GHG emission flux (mg m^−2^ h^−1^/μg m^−2^ h^−1^), ρ is the gas density (g m^−3^) at 273 K and 0.101 MPa pressure, H is the height of the chamber (m), $\frac{∆C}{∆t}$ is the rate change in gas concentration inside the chamber (g m^−3^ min^−1^), T is the air temperature (K) inside the chamber, and *k* is the time conversion factor (min h^−1^). A positive or negative *F* value indicates that soil is the source or sink of the GHG, respectively. 

The amount of N_2_O, CO_2_, and CH_4_ emissions on the sampling day is calculated as follows:

(2) R=F×a×b×c

where *R* is the total amount of CO_2_, N_2_O, and CH_4_ emissions (kg ha^−1^), F is the GHG emission flux (mg m^−2^ h^−1^/μg m^−2^ h^−1^), *a* is the time conversion factor (h day^−1^), *b* is the area convention coefficient (10,000 m^2^ ha^−1^), and *c* is the weight conversion factor (10^6^ mg kg^−1^/10^9^ μg kg^−1^). Liner interpolation method was used to calculate the daily GHG emission between two sampling dates. 

#### 4.3.3. Carbon Dioxide Equivalent (CO_2_-eq) in Maize Life Cycle

The life cycle assessment (LCA) methodology was used in this study to quantify the GHG emissions during the whole maize production process. GHG emissions at the system boundary of maize planting in the LCA were represented by Carbon Dioxide Equivalent (CO_2_-eq), divided into direct and indirect emissions, and calculated as follows:

(3) $$CE=CE_{direct}+CE_{indirect}$$

where *CE_direct_* (kg CO_2_-eq ha^−1^) and *CE_indirect_* (kg CO_2_-eq ha^−1^) are the direct and indirect GHG emissions in the maize life cycle, respectively. A positive *CE* value indicates a net C source, and a negative value reflects a net C sink.

The direct GHG emissions are from maize farmland under different N fertilizer treatments, including CO_2_, N_2_O, and CH_4_.

(4) $$CE_{direct}=R_{{\mathrm{CO}}_{2}}+R_{N_{2}O}\times 265+R_{{\mathrm{CH}}_{4}}\times 28$$

where $R_{N_{2}O}$ is the total amount of N_2_O emissions (kg ha^−1^), and $R_{{CH}_{4}}$ is the total amount of CH_4_ emissions (kg ha^−1^). The factors 265 and 28 are the default molecular GWPs of N_2_O and CH_4_, respectively, for a 100-year time horizon [58].

The indirect GHG emissions comprise farm management practices (sowing, tillage, irrigation, and harvesting) as well as the production and transportation of agricultural materials (seeds, fertilizer, and pesticides).

(5) $$CE_{indirect}=\sum_{i=1}^{n}CE_{i}=\sum_{i=1}^{n}m_{i}\beta_{i}$$

where *m_i_* and *β_i_* are the agricultural inputs and relevant emission coefficients of the indirect GHG emissions [59], as shown in Table 3. 

As widely used to evaluate the efficiency of the crop system that produces yield and GHG emissions, carbon emission efficiency (*CE_Y_*) is the carbon equivalent per unit crop yield [60]. The *CE_Y_* was calculated as:

(6) $$CE_{Y}=\frac{CE}{Y}$$

where *CE_Y_* is the GHG emissions per unit yield and *Y* is the crop yield (kg ha^−1^).

**Table 3:** Carbon equivalent emissions for agricultural inputs used in the field experiment.

Agricultural Inputs	Emission Coefficients (kg C per Unit Input)	References
N	2.116	Chen et al., 2015 [49]
P_2_O_5_	0.636	Chen et al., 2015 [49]
K_2_O	0.18	Chen et al., 2015 [49]
Herbicide	6.3	Lal, 2004 [61]
Insecticide	5.1	Lal, 2004 [61]
Diesel fuel	0.94	Lal, 2004 [61]
Electricity for irrigation	0.31	Yuan et al., 2006 [62]
Seed	1.05	West and Marland, 2002 [63]

#### 4.3.4. Agricultural Economy in Maize Life Cycle

The LCA method was also used for agricultural economic analysis by considering all agricultural inputs and outputs in the maize growth process. The agricultural inputs were the same as in the calculation of CO_2_ Equivalent, i.e., the costs of farm management practices and materials production and transportation in maize planting, and the output was the economic benefits generated by the maize yield. The unit prices of agricultural inputs are plotted in Table 4. The maize prices on the domestic market from 2018 to 2020 were 291.0, 336.8, and 321.5 US$ t^−1^.

In addition, as the world carbon emissions trading market was being established, we assumed that the agricultural economic analysis should comprise the cost of carbon trading, which was calculated as follows:

(7) $$\mathrm{Cost}\;\mathrm{of}\;\mathrm{carbon}\;\mathrm{trading}\;(\mathrm{US}\$)=CE\;({\mathrm{tCO}}_{2}{\text{-}\mathrm{eq}}^{-1})\times 9.4\;({\mathrm{US}\$\;t}^{-1})$$

The average carbon trading price was 9.4 US$ t^−1^, which was obtained from the China Beijing Green Exchange Institute (https://www.cbeex.com.cn/, accessed on 15 November 2022).

The agricultural net profit (ANP) of summer maize was the difference between agricultural outputs and inputs (including the cost of carbon trading), and the cost-benefit ratio was the ratio of total agricultural outputs to inputs.

### 4.4. Statistical Analysis

Experimental data were compiled using Excel 2021. The effects on various parameters were analyzed by analysis of variance (ANOVA) for treatments using SPSS 20.0 (SPSS software China, Beijing, China). The mean values of various treatments were tested for statistical significance at a 5% (*p* < 0.05) level of probability using Duncan’s multiple range test.

## 5. Conclusions

The 4-year field experiment conducted in a maize field in the North China Plain showed that different N management practices had different impacts on annual GHG emissions and economic profit. The yield under the SU and ER treatments had no significant differences in all four years. Under the same nitrogen application rate, adding inhibitors significantly mitigated direct emissions resulting from the decreases in N_2_O. Nitrogen fertilizer contributed to direct and indirect GHG emissions; thus, the HF treatment produced significantly higher emissions from a whole-life cycle perspective. A non-significant difference in ANP was found for the SU and ER treatments, indicating that adding fertilizer inhibitors did not affect farmers’ income while reducing emissions. These results suggest a feasible pathway for maintaining yield while minimizing GHG emissions and maximizing ANP.

## Figures and Tables

**Table 1:** Yield and yield components under different treatments.

Year	Treatment	Number of Ears (667 m^−1^)	Number of Kernels per Ear (ear^−1^)	1000-Kernals Weight (g)	Yield (kg ha^−1^)	Yield Increase Rate Compared with BF (%)
2017	BF ^1^	4724.6 a ^2^	456.2 b	348.7 a	10,961.9 b	-
	SU	4594.9 a	500.4 ab	366.6 a	12,376.8 a	12.9
	ER	4706.0 a	520.7 a	370.4 a	13,005.6 a	18.7
	HF	4446.7 a	491.1 ab	374.1 a	12,042.5 ab	9.9
2018	BF ^1^	4705.0 a	465.3 b	353.1 a	9797.9 b	-
	SU	4817.2 a	521.7 a	375.6 a	12,131.9 a	23.8
	ER	4779.8 a	525.0 a	367.3 a	11,761.3 a	20.0
	HF	4668.6 a	511.2 ab	375.1 a	11,504.8 a	17.4
2019	BF ^1^	4451.5 a	536.7 a	348.7 a	10,646.0 b	-
	SU	4444.7 a	571.4 a	375.7 a	12,166.1 a	14.3
	ER	4348.6 a	564.2 a	374.7 a	11,714.8 ab	10.0
	HF	4506.4 a	554.0 a	341.1 a	10,869.8 b	2.1
2020	BF	4417.2 a	479.7 b	328.5 b	9212.8 b	-
	SU	4430.9 a	538.4 a	363.9 a	10,813.6 a	24.0
	ER	4492.7 a	521.4 ab	359.7 ab	10,635.7 a	20.7
	HF	4444.7 a	523.6 ab	345.1 ab	10,246.6 a	15.0
Average	BF	4517.1 a	484.5 a	343.8 a	10,153.7 b	-
	SU	4508.6 a	532.8 a	367.7 a	11,867.2 a	18.7
	ER	4493.3 a	532.8 a	364.1 a	11,777.7 a	17.4
	HF	4476.5 a	520.7 a	364.5 a	11,155.6 ab	11.1

^1^ BF: basic N fertilizer treatment; SU: suitable utilization of fertilization; ER: emission reduction treatment, same fertilization as SU with a nitrification inhibitor and urease inhibitor; HF: higher fertilization than SU. ^2^ Different letters following values denote a significant difference at *p* < 0.05 based on Duncan’s new multiple-range test d two treatments with the same letter (a, b, c, etc.) indicate an insignificant difference.

**Table 2:** Application rates of fertilizers in different treatments during maize season in the long-term experiments (kg ha^−1^).

Treatment	N	P_2_O_5_	K_2_O	Others
BF ^a^	23.4	60	90	-
SU	180	120	105	-
ER	180	120	105	DCD ^b^: 21.75, HQ: 0.9
HF	300	180	210	-

^a^ BF: basic N fertilizer treatment; SU: suitable utilization of fertilization; ER: emission reduction treatment, same fertilization as SU with a nitrification inhibitor and urease inhibitor; HF: higher fertilization than SU. ^b^ DCD, dicyandiamide, a nitrification inhibitor; HQ, hydroquinone, a urease inhibitor.

**Table 4:** The amounts and unit prices for agricultural inputs used in the field experiment.

Agricultural Inputs	Amount	Unit Price
N	As shown in Table 2	367.4 US$ t^−1^
P_2_O_5_	As shown in Table 2	765.5 US$ t^−1^
K_2_O	As shown in Table 2	459.3 US$ t^−1^
Herbicide	2.0 kg ha^−1^	1148.3 US$ t^−1^
Insecticide	0.6 kg ha^−1^	888.0 US$ t^−1^
Diesel fuel	68 kg ha^−1^	1.2 US$ kg^−1^
Electricity for irrigation	750 kWh ha^−1^	0.1 US$ kWh^−1^
Seed	28.2 kg ha^−1^	3.1 US$ kg^−1^
Nitrification inhibitor	As shown in Table 2	1.2 US$ kg^−1^
Urease inhibitor	As shown in Table 2	1.7 US$ kg^−1^

## Data Availability

All data generated or analyzed during this study are included in this published article.
